# Circadian misalignment potentiates blood-brain barrier disruption and mitochondria dysregulation in Zika virus infection

**DOI:** 10.21203/rs.3.rs-6298126/v1

**Published:** 2025-04-22

**Authors:** Timea Teglas, Silvia Torices, Anne Caroline Marcos, Bogusława Orzechowska-Wylęgała, Michal Toborek

**Affiliations:** 1 Department of Biochemistry and Molecular Biology, University of Miami Miller School of Medicine, Miami, FL, USA; 2 Department of Pediatric Otolaryngology and Surgery of Head and Neck, Medical University of Silesia, Katowice, Poland; 3 Institute of Physiotherapy and Health Sciences, The Jerzy Kukuczka Academy of Physical Education, Katowice, Poland

**Keywords:** Zika virus, blood-brain barrier, tight junctions, mitochondria, circadian rhythm

## Abstract

**Background:**

Zika virus (ZIKV) is a mosquito-borne Flavivirus with a strong affinity for the central nervous system (CNS). After infection, ZIKV can cross the blood-brain barrier (BBB) and reach the CNS, causing potential harm to both adult and developing brains.

**Methods:**

The current study aims to evaluate how dysregulated circadian rhythms can affect brain infection by ZIKV, as biorhythms regulate essential physiological processes and disrupted circadian clock can contribute to the pathogenesis of multiple disorders. Both ZIKV infection and circadian rhythm alterations have been related to the disruption of the BBB integrity by modulating the expression of the tight junction (TJ) proteins, however, the input of circadian misalignment on ZIKV infection has never been studied in the literature.

**Results:**

Infection of brain endothelial cells with ZIKV selectively impacted endothelial permeability and dysregulated the expression of TJ and mitochondrial proteins. Importantly, these effects were potentiated by silencing *Bmal1*, a critical circadian rhythm gene. These results were then confirmed *in vivo* in *Bmal1* endothelial cell-specific knockout mice, which were infected with ZIKV at 10^5^ PFU (plaque-forming unit) by retro-orbital infusion. ZIKV infection resulted in a marked decrease in claudin-5, occludin, JAM-3, and ZO-1 expression levels in these mice. In addition, ZIKV affected the expression of FIS1 protein levels and the respiratory complexes of II, III, and IV in mice lacking *Bmal1* expression in endothelial cells.

**Conclusions:**

Findings from this study contribute to a better understanding of the impact of circadian misalignment on the pathology of ZIKV infection in the adult brain.

## Graphical abstract.

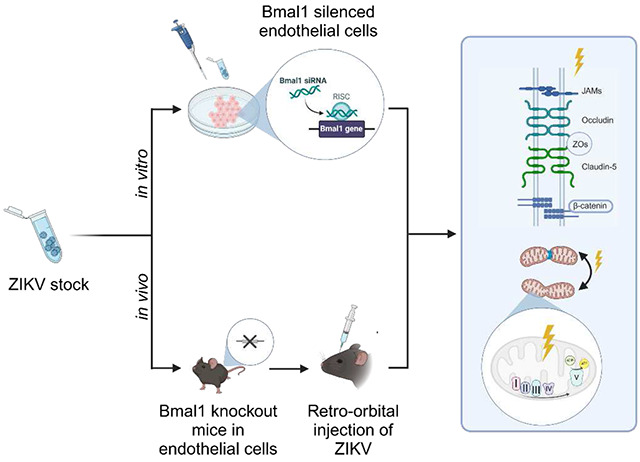

Endothelial cells and endothelial cell-specific Bmal1 knock-out mice were subjected to Zika virus infection. Bmal-1 expression deficiency potentiated the severity ZIKV infection and contributed to endothelial barrier dysfunction and dysregulation of mitochondrial proteins.

## Background

Mosquito-borne Flavivirus, Zika virus (ZIKV), belongs to the Flaviviridae family and contains a positive single-stranded RNA. It was first isolated from nonhuman primates in 1947 and from mosquitoes in 1948 in Africa [1]. ZIKV, once regarded as causing only mild infections, has now become one of the most studied viruses worldwide due to its complex and profound impact on human health [2]. ZIKV infection constitutes an emerging challenge to public health as there are no specific therapeutics or vaccine for treatment or prevention. The preventive measures are indirect and include use a bed net, water treatment tabs, repellents, and sprays to avoid mosquito bites [3,4].

Upon infection, ZIKV can cross tissue barriers, including the placental barrier and the blood-brain barrier (BBB) [5,6]. The BBB is a critical regulator of CNS homeostasis, with its integrity being ensured by tight junction (TJ) proteins, which form complexes that serve as a primary barrier to prevent molecules from diffusing across the BBB [7]. Several studies have shown that ZIKV infects the developing brain [8,9], with the most severe outcomes being related to the neuropathology of the human fetal brain. On the other hand, only limited studies were performed to examine ZIKV infection in the adult brain. A wide spectrum of neurological disorders can develop in adult ZIKV-infected patients, including Guillain-Barré syndrome, encephalitis, meningoencephalitis, myelitis, sensory neuropathies, optic neuropathy, and seizures. Stroke and epilepsy are also complications of ZIKV infection in adults [10–12]. Collectively, these studies confirm that ZIKV exhibits a strong affinity for the mature CNS, implying that the virus has potential to harm not only to the developing but also to the adult brains. ZIKV infections are typically accompanied by dysregulations of the TJ proteins. It was demonstrated that an early stage of ZIKV infection can selectively disrupt TJ protein expression on brain microvessels and BBB permeability in mice [13]. These results were confirmed in another study demonstrating that infections with ZIKV can reduce both occludin and DLG1 adherent junction protein levels in a strain-dependent manner [14].

The role of circadian systems in the regulation of viral infection is another emerging area of research, supported by experimental and clinical evidence reporting on the interplay between circadian disruption and viral infections [15]. Circadian genes influence the hormonal, metabolic, and immune systems, impacting neurological disorders, stress responses, and the gut microbiome [16–18]. Importantly, circadian clock components regulate the host immunity which impacts virus infection and flavivirus replication [19]. Nevertheless, there is no information on whether ZIKV infection can alter the circadian clock in the adult brain as it was shown *in vitro* in human neuronal cells [20].

Mitochondria actively regulate inflammatory and innate immune responses and viral infections that are known to exploit mitochondria-mediated cellular processes [21–23]. For example, it has been demonstrated that ZIKV infection of trophoblast cells can manipulate mitochondrial dynamics and mitophagy [24]. In addition, abnormal mitochondrial fragmentation via rebalancing mitochondrial dynamics of fission and fusion proteins was observed in ZIKV infection *in vitro* [25]. Finally, mitophagy activation targeting PINK1 was identified as a potential therapeutic target in ZIKV infection [26]. Based on these literature reports, the aim of this study was to evaluate the influence of dysregulation of circadian rhythms on brain infection by ZIKV with a mechanistic focus on BBB integrity and mitochondrial functions.

## Materials and Methods

### Cell Cultures

Vero cells (African green monkey kidney epithelial cells) were obtained from the American Type Culture Collection (ATCC, #CCL-81) and cultured in DMEM containing GlutaMAX (Thermo Fisher Scientific, #35050061) and supplemented with 10% heat-inactivated fetal bovine serum (FBS) (ATCC, #30-2020) and 1% Penicillin/Streptomycin (Thermo Fisher Scientific, #15140122) at 37°C with 5% CO_2_. The human brain capillary endothelial cell line hCMEC/D3 was cultured in Endothelial Cell Basal Medium-2 (Lonza, EBM-2, #CC-3156 and EGMTM-2 Endothelial SingleQuots^™^ Kit, #CC-4176) supplemented with vascular endothelial growth factor, insulin-like growth factor 1, epidermal growth factor-B, fibroblast growth factors, hydrocortisone, ascorbic acid, GA-1000, heparin, and 2.5% of fetal bovine serum. The cells were grown on rat tail collagen type I-coated (Sigma-Aldrich, #C3867-1VL) dishes at 37°C with 5% CO_2_.

### Virus production and titration

Zika virus strain R103451 (Honduras 2015) was obtained from ATCC (#VR-1848) and propagated in Vero cells at a multiplicity of infection (MOI) of 0.01 in serum-free DMEM for 2 h. After incubation, the virus inoculum was removed, and pre-warmed complete DMEM was added to the cells. Supernatants were collected four days post-infection (dpi), clarified by 0.45 μm cellulose acetate filter (Corning, #430314), and concentrated using centrifugal filters with 30 kDa molecular weight cut-off (EMDMilipore, #UFC9030). HEPES buffer (Gibco, #15-630-08) was added to the concentrated stock to prevent the acidification of the virus following freeze and thawing. The virus was tittered (10^−1^ to 10^−6^) by plaque assay using Vero cells with a 0.7% agarose overlay (Sigma Aldrich, #A9414). As a control, mock-infected Vero cells were subjected to the same procedure. Foci of plaques were detected at 3 dpi, following fixation with methanol: acetic acid (3:1 ratio) solution and staining with 0.1% crystal violet.

### Gene silencing

Brain endothelial cells plated in 6-well plates were subjected to siRNA transfection to silence the *Bmal1* gene upon reaching 80% confluence. The transfection mix was prepared in Opti-MEM (Invitrogen, #11058021) medium containing Silencer Negative Control (Ambion, #AM4611) and Bmal1 siRNA (Dharmacon, #L-012977-00-0005), along with Lipofectamine RNAiMAX according to the manufacturer’s protocol (Invitrogen, #13778-075). The final concentration of siRNA and Lipofectamine added to the cells was 75 nM and 7.5 μL/mL, respectively. Cells were incubated in the presence of a transfection mixture for 6 h, and then with EBM-2 complete medium for an additional 18 h. Cells were infected with ZIKV 24 h after the start of transfection and then maintained in culture for 2 days.

### RT-qPCR

Total RNA was isolated from 350 μL of half brain tissue homogenates or 100 μL of cell culture lysates, using a RNeasy kit (Qiagen, #74124). RT-qPCR of cell culture lysates and brain tissue total RNA samples was performed using primers specific for ZIKV evaluated in the present study and were purchased from Integrated DNA Technologies (5′-CCG CTG CCC AAC ACA AG-3′ and 5′-CCA CTA ACG TTC TTT TGC AGA CAT-3′, probe 5′-6FAM-AGC CTA CCT TGA CAA GCA GTC AGA CAC TCA A-IABkFQ-3′). In addition, the following primers were purchased from Thermo Fisher Scientific: Bmal1 (Hs00154147_m1), β-catenin (#Hs00355045_m1), Claudin-5 (#Hs00533949_s1), Occludin (#Hs05465837_g1), Jam-3 (#Hs00230289_m1), ZO-1 (#Hs01551871_m1). The qScript XLT 1-Step RT-qPCR ToughMix (Quantabio) was used as a reaction mix, and the analyses were performed using the 7500 Real-Time PCR System (Thermo Fisher Scientific). mRNA expression levels of genes of interest were normalized to the expression levels of human GAPDH or mouse hemoglobin beta chain complex (HBB) (Thermo Fisher Scientific, VIC).

### Immunoblotting

Protein fractions from cell culture lysates and isolated brain microvessels were extracted with RIPA buffer supplemented with protease inhibitors and 1% Triton X-100 to inactivate ZIKV. The homogenates were incubated for 30 min with gentle shaking on ice, then centrifuged at 15,000 x g for 15 min at 4°C. Protein concentrations were measured from the supernatant by Pierce BCA Protein Assay Kit (Thermo Fisher Scientific). The samples (15 μg protein/well) were loaded on sodium dodecyl sulfate (SDS) polyacrylamide 4–20% or 8-16% ready gels (BioRad) and electrotransferred to a 0.2 μm nitrocellulose membrane using a transfer pack system (BioRad). The blots were incubated overnight at 4°C with the primary antibodies listed in Supplementary Table 1. After being washed three times with TBST, the membranes were incubated with mouse or rabbit secondary antibodies (LI-COR, #926-32210, #926-68070, #926-32211, #926-68071) at 1:20,000 for 1 h at 4°C. The protein bands were analyzed using the Licor CLX imaging system and the Image Studio 4.0 software (LI-COR, Lincoln, NE, USA).

### Immunostaining of endothelial cells

Immunostaining was performed on brain endothelial cells cultured on a glass coverslip in 6-well plates (Thermo Fisher Scientific). Cultures were carefully washed twice with PBS and fixed with 4% paraformaldehyde (Santa Cruz Biotechnology). The cells were permeabilized with 0.1% Triton-X solution in PBS, followed by blocking with 3% bovine serum albumin (BSA) in PBS for 1 h at room temperature, and overnight at 4°C with rabbit antibodies against the ZIKV NS-1 protein (GeneTex; #GTX133307) (diluted 1:100 in 4 % BSA in PBS). Anti-rabbit Alexa-488 (Thermo Fisher Scientific) diluted 1:300 in 1% BSA in PBS was used as a secondary antibody, and the slides were mounted with Vectashield HardSet Antifade Mounting Medium with DAPI (Vector Labs). Confocal microscopy (FV3000, Olympus, Tokyo, Japan) was used to capture images.

### TEER measurement and permeability assay

Transendothelial electrical resistance (TEER) as an indicator of the barrier integrity, was estimated by using an automated impedance-based cell monitoring CellZscope2 system (NanoAnalytics, Germany). Endothelial cells grown on the CellQART 24-well cell culture inserts (Sterlitech, Cat #9320412), were seated in stainless steel electrode pots (wells) screwed into the base unit of the instrument. Cell culture inserts without cells were constructed as a blank. Data was monitored and analyzed by the provided CellZscope2 software. For data presentation, the relative TEER (% of control) values were calculated.

Permeability across endothelial monolayers was performed using 24-well 0.4 μm pore PET transwell plates (Corning). Brain endothelial cells were seeded at 1 × 10^5^ cells per insert in complete EBM-2 media. After 2 days, the cells were transfected with Bmal1 (or control) siRNA followed by ZIKV infection at MOI 0.01. At 2 dpi, the cells were washed with HBSS buffer two times, and the media was replaced with phenol red-free EBM media in both chambers. Fluorescently tagged 20 kDa dextran (Sigma-Aldrich, #FD20-250MG) was added to the upper chamber at a final concentration of 0.5 mg/mL. After 90 min of incubation, transendothelial movement of 20 kDa FITC-dextran was analyzed at 485 nm (Ex) and 525 nm (Em) by a fluorescence plate reader (Thermo Fisher Scientific, Waltham, MA, USA).

### Endothelial-specific conditional *Bmal1* knockout mice

The Bmal1^fx/fx^ (Bmal1^fx/fx^, B6.129S4(Cg)-Arn<tltm1Weit>/J) and Tek-Cre (B6.Cg-Tg(Tek-cre)1Ywa/J) mice were purchased from the Jackson Laboratory (#0076, #00886). We then crossed Bmal1^fx/fx^ mice with Tek-Cre transgenic mice to generate either conditional endothelial cell Bmal1 knockout mice (Bmal1^ECKO^) or controls (WT). The scheme of breeding is represented in Supplementary Figure 1. All animals were maintained under 12 h light/dark conditions and ad libitum food and water intake. All experiments were performed when the animals aged between 6 and 8 months. All animal experiments were consistent with the National Institutes of Health (NIH) guidelines and all procedures were achieved following the protocols approved by the University of Miami Institutional Animal Care and Use Committee (IACUC).

The genetic makeup of mice was confirmed by genotyping. Briefly, genomic DNA was isolated from mouse ear tissue using a 50 mM NaOH solution and warmed at 90°C for 1 h. Then, 1 M Tris (pH 8.0) was added to the samples, mixed by vortexing, and centrifuged for 1 min at 10,000 rpm. The genomic DNA samples were stored at −20°C until the next step of the method. The DNA was amplified with a KAPA2G Robust HotStart PCR Kit (Fischer Scientific, #50-196-5197) following the protocol provided by the manufacturer using C100 Thermal Cycler (Biorad, Hercules, California, United States). The primers were purchased from Integrated DNA Technologies (transgene forward: 5’-GCG GTC TGG CAG TAA AAA CTA TC-3’, transgene reverse: 5’-GTG AAA CAG CAT TGC TGT CAC TT-3’, internal positive control forward: 5’-CTA GGC CAC AGA ATT GAA AGA TCT-3’, internal positive control reverse: 5’- GTA GGT GGA AAT TCT AGC ATC ATC C-3’ for Tek-Cre; and forward: 5’-ACT GGA AGT AAC TTT ATC AAA CTG-3’, reverse: 5’-CTG ACC AAC TTG CTA ACA ATT A-3’ for Bmal1^fx/fx^). For the agarose gel electrophoresis, 1% agarose gel was prepared with Syber green (1:10,000) and 20 μL samples and 5 μL 100bp DNA ladder (Invitrogen, #10488058) was loaded and run in 1 x TAE running buffer at 90V for 1 h. UV Transilluminators visualized the separated DNA.

### Mouse infection with ZIKV

Mice were anesthetized with isoflurane inhalation, then infused intravenously via retro-orbital venous sinus with 100 μL of 10^5^ plaques forming units (PFU) of ZIKV stock or PBS (mock infection). At 2 dpi, animals were euthanized and transcardiac perfusion was employed with normal saline. Brain tissues were harvested, snap-frozen in liquid nitrogen, and stored at −80°C.

### Brain microvessel isolation

Briefly, brain tissue was cut into small pieces with a scissor and homogenized in a homogenization buffer containing 102.0 mM NaCl, 4.7 mM KCl, 2.5 mM CaCl_2_, 1.2 mM KH_2_PO_4_, 1.2 mM MgSO_4_, 15 mM HEPES, 25 mM NaHCO_3_, 10 mM glucose, and 1 mM Na pyruvate; pH 7.4 with complete inhibitor cocktail (Roche, #11697498001) using a Teflon grinder and Con-Torque Tissue Homogenizer (Eberbach, Van Buren Charter Township, MI). The homogenates were filtered through a 300 μm nylon mesh filter (Thermo Fisher Scientific, #146424), gently agitated with 26 % dextran for 2 min, and centrifuged at 5800 x g for 30 min at 4 °C. The pellet was resuspended in an ice-cold homogenization buffer solution, passed through a 120 μm nylon mesh filter (Merck, #NY2H09000), and centrifuged at 1500 x g for 10 min at 4 °C. The pellet was resuspended in RIPA buffer for protein extraction.

### Fluorescent immunohistochemistry of mouse brain

After fixation in 4% PFA, the brain samples were incubated for 2 days in a 30% sucrose solution and placed in an O.C.T block (Tissue-Tek, #4583). For cryostat sectioning, 12 μm-thick sections were prepared on Superfrost slides (VWR, #48311-703) using Cryostar NX70 (Thermo Fischer Scientific) at −20°C. The tissue slides were rehydrated three times with PBS for 5 min. For antigen retrieval, 1% SDS solution in PBS was used for 5 min. After washing, the tissue slides were permeabilized in 0.1% Triton-X solution in PBS for 10 min. For blocking, NATS solution (20% FBS, 0.5% Tween-20 in PBS) and mouse IgG Blocking Solution (Invitrogen, #R37621) was employed for 1 h at room temperature. Then, Bmal1 (#NBP2-02544, mouse, 1:100) or CD31 Alexa 488 (#AB307133, 1:300) primary antibodies were used for overnight incubation at 4°C. The next day, slides were washed with PBST and incubated with Alexa Fluor 594 secondary antibodies (Thermo Fisher Scientific, mouse) for 2 h at room temperature. The slides were mounted with Vectashield HardSet Antifade Mounting Medium with DAPI (Vector Labs). Confocal microscopy (FV3000, Olympus, Tokyo, Japan) was used to capture images.

### Statistical analysis

The results were analyzed by Student’s *t*-test, one-way or two-way Analysis of Variance (ANOVA). P-values ≤ 0.05 were considered to be significant. Results are expressed as means ± Standard Error of Mean (SEM). The GraphPad Prism software (GraphPad, San Diego, CA, USA) was used for all statistical analyses.

## Results

### ZIKV infection of brain endothelial cells elevates Bmal1 protein expression

**Figure 1.**
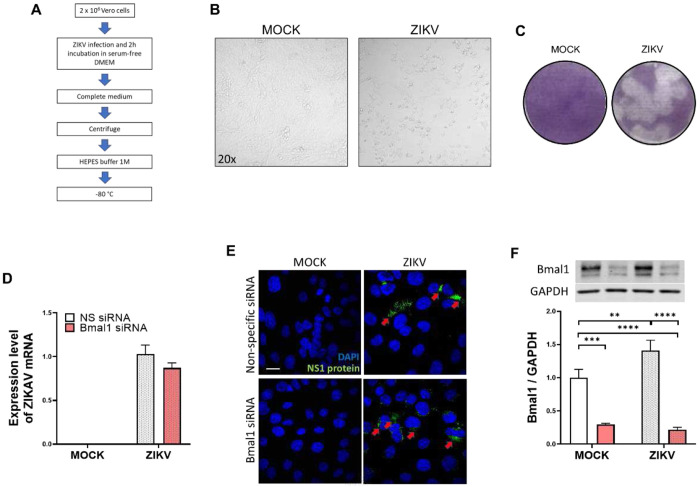
ZIKV production, titration, and endothelial infection *in vitro*. **(A**) Schematic diagram of ZIKV production. Zika virus strain R103451 (Honduras) was propagated in Vero cells at a multiplicity of infection (MOI) of 0.01 in serum-free DMEM for 2h. After incubation, the virus inoculum was removed, and complete DMEM was added to the cells. Supernatants were collected four days post-infection (dpi). HEPES buffer was added to the stock to prevent the acidification of the virus following freeze and thawing. (**B**) Cytopathic effects of ZIKV infection in Vero cells at 4 dpi. Mock infection serves as a comparison. Note changes in cell shapes and floating cells, indicating cell death. (**C**) ZIKV was tittered (10^−1^ to 10^−6^) by plaque assay using Vero cells with a 0.7% agarose overlay. As a control, mock-infected Vero cells were subjected to the same procedure. Foci of plaques were detected at 3 dpi, fixed with methanol: acetic acid (3:1 ratio), and stained with 0.1% crystal violet. (**D**) The expression of ZIKV mRNA in mock and ZIKV-infected (MOI 0.01) wild-type or *Bmal1*-silenced brain endothelial cells at 2 dpi. NS, non-specific. (**E**) Representative images of ZIKV-specific NS1 protein (green) in control and/or *Bmal1*-silenced brain endothelial cells infected as in (D). Blue, nuclear staining with Hoechst 33342. (**F**) Bmal1 protein expression in *Bmal1*-silenced or NS-transfected brain endothelial cells infected as in (D). Upper panel, immunoblots of Bmal1 protein. Lower panel, quantitative protein expression data. The results were normalized to GAPDH levels. Values are mean ± SEM; n=3. *p<0.05. Scale bar: 20 μm.

Stock solution of ZIKV was generated by infecting Vero cells with ZIKV at MOI of 0.01 (Figure 1A) for 2 h in serum-free media, which was then replaced with fresh growth medium. At 4 dpi and upon the appearance of the cytopathic effects (Figure 1B), the supernatants were collected, centrifuged through an MCE filter, and stored at −80°C in HEPES buffer. Generated ZIKV was titrated by plaque assay using Vero cells with a 0.7% agarose overlay as described earlier (Leda et al., 2019). Plaque numbers were counted to calculate the plaque-forming units (PFU/mL) (Figure 1C).

We then evaluated the impact of circadian disruption by *Bmal1* gene silencing on ZIKV infection of brain endothelial cells. Briefly, brain endothelial cells cultured on 6-well plates were transfected with *Bmal1* siRNA, following infection with ZIKV at MOI 0.01. ZIKV mRNA levels were then assessed in cell lysates at 2 dpi. ZIKV infected both wild-type and Bmal1-silenced brain endothelial cells (Figures 1D and 1E). Moreover, this infection was productive as it resulted in the production of ZIKV-specific protein, NS1 (Figure 1E). Silencing of *Bmal1* did not affect ZIKV mRNA levels as compared to transfection with non-specific siRNA (Figure 1D). Interestingly, ZIKV infection elevated the Bmal1 protein levels in brain endothelial cells as compared to Mock infection (Figure 1F).

### Selective impact of *Bmal1* silencing and/or ZIKV infection on the expression of TJ genes and proteins in endothelial cells

**Figure 2.**
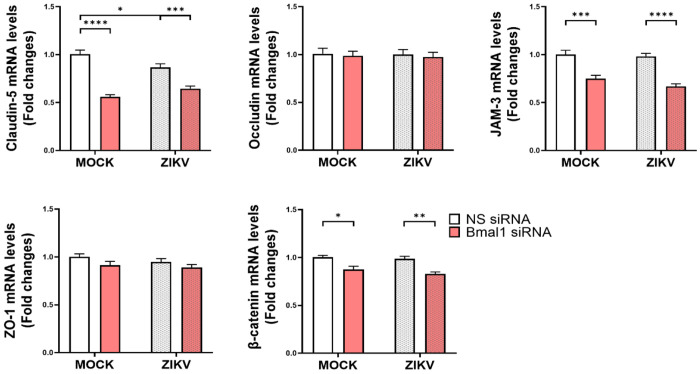
Impact of *Bmal1* silencing and/or infection with ZIKV on the expression of TJ genes. Brain endothelial cells were transfected with 75 nM *Bmal1*-specific or non-specific (NS) siRNA, followed by infection with ZIKV as in Figure 1D. *Claudin-5*, *occludin*, *JAM-3*, *ZO-1*, and β-*catenin* mRNA levels were measured by RT-qPCR, with GAPDH mRNA expression serving to normalize the results. Values are mean ± SEM; n=6 per group; *p<0.05, **p<0.01, ***p<0.001, ****p<0.0001.

In the next series of experiments, we focused on the impact of ZIKV infection and/or *Bmal1* silencing on the expression of TJ genes and proteins, such as claudin-5, occludin, zona occludens (ZO-1), and junctional adhesion molecule-3 (JAM-3). We also evaluated β-catenin expression, which is the core component of the cadherin complex of cell adhesion junctions. mRNA expression of *claudin-5*, *JAM-3*, and *β-catenin* were decreased by *Bmal1* silencing in both ZIKV and mock-infected groups. In contrast, mRNA levels of *occludin* and *ZO-1* were not altered by *Bmal1* silencing as compared to cells transfected with non-specific siRNA (Figure 2). In cells with normal expression of the *Bmal1* gene, infection with ZIKV at 2 dpi significantly decreased *claudin-5* mRNA levels (Figure 2).

**Figure 3.**
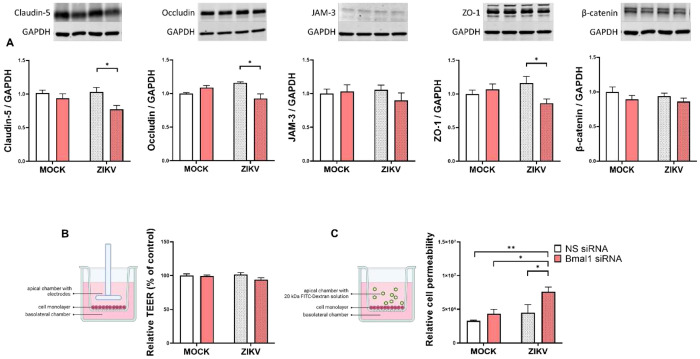
Impact of the *Bmal1* silencing and/or infection with ZIKV on the expression of TJ protein and endothelial barrier integrity. Cells were treated and infected as in Figure 1D. (**A**) Claudin-5, occludin, JAM-3, ZO-1, and B-catenin protein levels were measured by immunoblotting and normalized to GAPDH levels. Upper panels, representative immunoblots of TJ proteins. Lower panels, quantified protein expression data. Values are mean ± SEM; n=6; *p<0.05. (**B-C**) Endothelial barrier function. Brain endothelial cells were grown to confluency on the 0.4 μm membrane in the upper compartment of the CellQART 24-well culture system. Cell culture inserts without cells were constructed as a blank. Cells were transfected with *Bmal1* or NS siRNA, and infected with ZIKV. Data was monitored and analyzed using the provided CellZscope2 software. (**B**) The relative TEER values (% of control) at 2 dpi. Left panel, schematic representation of the method; Right panel, quantified data. (**C**) The transendothelial passage of 20 kDa FITC-dextran measured for 90 min at 2 dpi. Fluorescence intensity (A.U.) of FITC-dextran in the lower chamber was measured as a marker of endothelial integrity. Left panel, schematic representation of the method; Right panel, quantified data. Values are mean ± SEM; n=5-6; *p<0.05, **p<0.01.

Because gene expression changes can be only transitional, we also evaluated the impact of *Bmal1* silencing and/or ZIKV infection on TJ protein expression (Figure 3A). ZIKV infection significantly decreased claudin-5, occludin, and ZO-1 protein levels in cells with silenced *Bmal1* gene. In contrast, ZIKV did not affect expression of these proteins in cells transfected with non-specific siRNA; i.e., cells with regular expression of the *Bmal1* gene. These effects were specific as silencing of the *Bmal1* gene and/or infection with ZIKV did not affect JAM-3 and β-catenin protein expression.

### Impact of *Bmal1* silencing and/or ZIKV infection on endothelial barrier function

The impact of *Bmal1* silencing and/or ZIKV infection on endothelial integrity was assessed by measuring transendothelial electrical resistance (TEER) and transendothelial transfer of 20 kDa FITC-dextran. For both assays, endothelial cells were seeded in the 24-well, 0.4 μm pore PET transwell plates in 1 x 10^5^ density. The cells were transfected with non-specific or *Bmal1* siRNA, and infected on the next day with ZIKV at MOI 0.01 for 2 days. Relative TEER values (% of control) were not altered as a result of *Bmal1* silencing and/or ZIKV infection (Figure 3B). Then, the media were changed to phenol red-free EBM and FITC-dextran was added to the insert at 0.5 mg/mL for 90 min. In contrast to the TEER values, the FITC-dextran transfer assay revealed higher endothelial permeability in *Bmal1*-silenced cultures infected with ZIKV (Figure 3C).

### Impact of *Bmal1* silencing and/or ZIKV infection on mitochondrial dysfunction in brain endothelial cells

**Figure 4.**
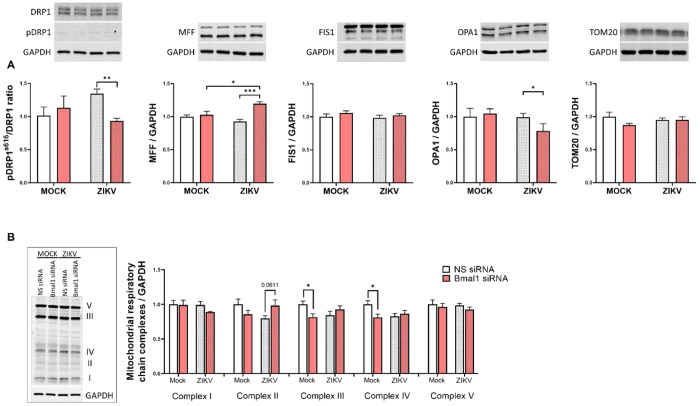
Impact of the *Bmal1* silencing and/or infection with ZIKV on mitochondrial dynamics. Brain endothelial cells were treated and infected as in Figure 1D. (**A**) Immunoblotting analysis for mitochondrial fission protein DRP1, pDRP1^s616^, MFF, FIS1, mitochondrial fusion protein OPA1, and mitochondrial marker TOM20. Upper panels, representative immunoblots. Lower panels, quantitative protein expression data. (**B**) Immunoblotting analysis of the expression of the mitochondrial respiratory chain complexes. Left panel, representative immunoblots. Right panel, quantitative protein expression data. The results were normalized to GAPDH levels. Values are mean ± SEM; n=5-6 per group. *p<0.05 and **p<0.01.

Next, we evaluated the expression of mitochondria-related proteins by focusing on proteins that regulate mitochondrial fission (MFF, DRP1, pDRP1^s616^, and FIS1), and fusion (OPA1). ZIKV infection in endothelial cells with silenced *Bmal1* gene upregulated MFF, indicating dysregulation of mitochondrial division. MFF controls mitochondrial fission by recruiting Drp1; therefore, it was surprising that the ratio of pDRP1^s616^/DRP1 decreased, while the expression of FIS1 did not change in *Bmal1*-silenced and ZIKV-infected cells. Moreover, the expression of OPA1, the key protein of mitochondrial fusion decreased in ZIKV-infected cells with silenced *Bmal1* gene. In addition to proteins that are important regulators in mitochondrial fission and fusion, we also evaluated the expression of the outer mitochondrial membrane protein, TOM20, which was not affected by *Bmal1* silencing and ZIKV (Figure 4A).

Analysis of the protein expression of mitochondrial respiratory chain complexes revealed a decreased levels of complexes III and IV in mock-infected and *Bmal1* silenced cells. In addition, the expression of complex II showed a strong tendency to increase in *Bmal1*-silenced and ZIKV-infected cells; however, the p-value 0.0611 did not reach a threshold for statistical significance (Figure 4B).

### ZIKV infection rate did not change in the brains of endothelial-specific conditional *Bmal1* knockout mice

**Figure 5.**
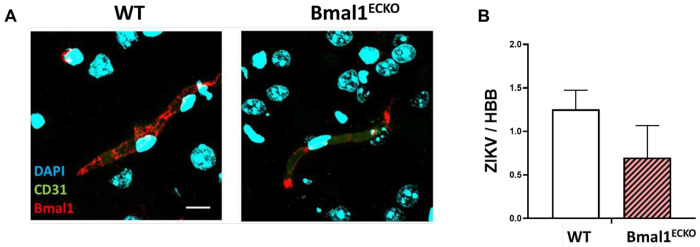
ZIKV infection of endothelial cell-specific conditional Bmal1-deficient mice (Bmal1^ECKO^). **A**) Representative confocal images of Bmal1 (red) and CD31 (green) immunoreactivity, and Hoechst 33342 (blue) staining in WT and Bmal1^ECKO^ brain microvessels. (**B**) ZIKV mRNA levels in infected, brain RT-qPCR positive WT and Bmal1^ECKO^ mouse brains. Mice were infected with ZIKV in 10^5^ PFU via retro-orbital injection. At 2 dpi, mice were sacrificed, and RNA was extracted from the brain tissues for RT-qPCR measuring. The results were normalized to HBB levels. Values are mean ± SEM; n=3-5. Scale bar: 20 μm

In order to address the importance of the BBB circadian dysregulation involved in brain infection by ZIKV, we generated endothelial cell-specific conditional *Bmal1* knock-out mice (Bmal1^ECKO^), with wild-type mice serving as controls (Supplementary Figure 1). Bmal1^ECKO^ mice displayed severely diminished Bmal1 expression in brain microvessels (Figure 5A). The residual Bmal1-positive immunostaining was likely derived from vascular pericytes and/or astrocyte end-feet.

Bmal1^ECKO^ and wild-type mice were infected with ZIKV with 10^5^ PFU or PBS (mock) for 2 days via retro-orbital injection, and ZIKV infection was then analyzed by RT-qPCR. Brain infection was detected in 21% of WT mice and 29% of Bmal1^ECKO^ mice that underwent ZIKV infusion. When analyzing infected brains, no differences in ZIKV mRNA levels were observed in Bmal1^ECKO^ mice as compared to wild-type controls (Figure 5D). Only the brains that tested positive for ZIKV infection were used in the following analyses.

### ZIKV infection decreased the expression of TJ proteins in microvessels of Bmal1^ECKO^ but not in control mice

**Figure 6.**
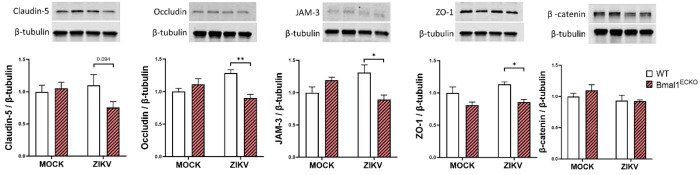
Impact of ZIKV infection on the expression of TJ proteins in WT and Bmal1^ECKO^ mice. Mice were infected as in Figure 5. (**A**) Claudin-5, occludin, JAM-3, ZO-1, and β-catenin protein levels were measured by immunoblotting and normalized to β-tubulin levels. Upper panels, representative immunoblots. Lower panels, quantitative protein expression data. Values are mean ± SEM; n=3-5; *p<0.05, and **p<0.01.

In the next series of experiments, brain microvessels from ZIKV-infected and mock-infected Bmal1^ECKO^ and WT mice were isolated and evaluated for the expression of TJ proteins by immunoblotting (Figure 6). Endothelial-specific knockout of the *Bmal1* gene did not affect the expression of TJ proteins studied in the present study in mock-infected mice. Importantly, ZIKV infection in Bmal1^ECKO^ mice resulted in a significant decrease in the expression of claudin-5, occludin, JAM-3, and ZO-1 proteins when compared to controls (Figure 6). A similar pattern was observed in claudin-5 protein expression; however, it did not reach statistical significance at p=0.094. In contrast, the expression of β-catenin protein did not show any significance in the studied mice.

### Impact of ZIKV infection on mitochondria dynamics and respiratory complexes in brain microvessels of Bmal1^ECKO^ mice

**Figure 7.**
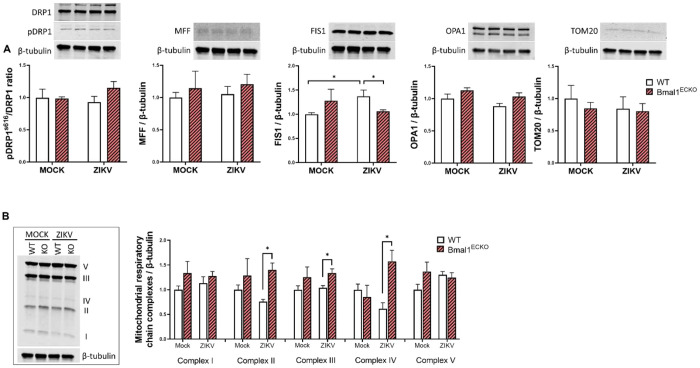
Impact of ZIKV infection on the mitochondrial dynamics in WT and Bmal1^ECKO^ mice. Mice were infected as in Figure 5. **(A)** Immunoblotting analysis of mitochondrial fission proteins pDRP1s^616^ and DRP1, MFF, and FIS1, mitochondrial fusion protein OPA1, and mitochondrial marker TOM20. Upper panels, representative immunoblots. Lower panels, quantitative protein expression data. (**B**) Protein expressions of the mitochondrial respiratory chain complexes. Left panel, representative immunoblots. Right panel, quantitative protein expression data. The results were normalized to β-tubulin levels. Values are mean ± SEM; n=3-5 per group. *p<0.05.

In the last series of experiments, we analyzed the impact of ZIKV infection on mitochondrial proteins in microvessels isolated from WT and Bmal1^ECKO^ mice. FIS1, the key protein in mitochondrial fission decreased in Bmal1^ECKO^ mice after ZIKV infection. In contrast, ZIKV infection upregulated FIS1 expression in WT mice. There was a tendency to upregulate the pDRP1^s616^/DRP1 ratio and OPA1 proteins by ZIKV in Bmal1^ECKO^ mice; however, they were not statistically significant. We also evaluated mitochondrial respiratory chain complexes in these mice. The results indicated a statistically significant ZIKV-related increase in Complex II, III, and IV protein expression in brain microvessels of Bmal1^ECKO^. In contrast, ZIKV infection of WT mice did not result in any changes in the expression of mitochondrial respiratory complexes (Figure 7B).

## Discussion

The present work aimed to study the impact of circadian rhythm disruption on ZIKV infection in adult brain. While ZIKV has evolved over the years, we analyzed the impact of the Honduras strain of ZIKV, representing the Asian lineage, which has been associated with Guillain-Barré syndrome and fetal microcephaly [12,27]. Regarding the employed model of circadian disruption, it should be noted that most circadian genes have functionally redundant paralogs, which requires that both genes of the pair have to be silenced to achieve arrhythmicity. For example, the deletion of *Clock* gene can be compensated for by its paralog neuronal PAS domain protein 2 (*Npas2*) [28]. However, *Bmal1* and its paralog, *Bmal2*, are an exception, and loss of Bmal1 downregulates Bmal2 expression, disrupting clock function and leading to arrhythmia [29]. Therefore, we silenced the *Bmal1* gene in *in vitro* studies and then used endothelial cell conditional *Bmal1* knockdown mice for animal experiments to verify the involvement of the brain endothelium and mitochondrial dynamics in ZIKV infection of the adult brain. Our results confirmed earlier reports that ZIKV can infect brain endothelial cells (Figure 1) and were consistent with observations that claudin-7 is required for optimal ZIKV replication in human endothelial cells [30]. However, there were no differences in infection efficiency when comparing cells with silenced *Bmal1* versus control cells with normal *Bmal1* expression. Interestingly, ZIKV infection resulted in an increase in the protein expression of Bmal1, supporting our choice of silencing *Bmal1* in the remaining series of experiments.

Literature data indicates that ZIKV infection can alter the integrity of tissue barriers by changing the expression of TJ proteins. For instance, it has been reported that ZIKV infection altered TJs protein expression and increased paracellular permeability in placentae syncytiotrophoblast cells [31]. Another *in vitro* study demonstrated that ZIKV can disrupt TJ proteins in human placenta trophoblast cells (JEG-3). Our laboratory demonstrated that the expression level of TJ proteins was disrupted in ZIKV-infected brain endothelial cells without affecting permeability [13]. These results corresponded with a report that found no changes in endothelial permeability in ZIKV-infected brain endothelial cells, even when a very high MOI (MOI=10) was employed (Chiu et al., 2020a). In contrast, ZIKV E protein was shown to dysregulate the expression of TJ and adherens junction proteins, resulting in increased endothelial permeability [32]. Animal studies have also observed disturbances in the expression of BBB proteins, such as β-catenin, occludin, and connexin-43 in the hippocampus of the rat [33].

To the best of our knowledge, the impact of circadian disruption on ZIKV-induced dysfunction of tissue barriers has never been evaluated in the literature. Notably, our novel findings indicated that *Bmal1* silencing can potentiate ZIKV-induced downregulation of selected TJ proteins, such as *claudin-5*, *JAM-3*, and *β-catenin*, confirming the dependency of selected TJ proteins on circadian regulation. Indeed, circadian rhythms have been shown to affect the expression of TJ genes and proteins. Previous research, including our own, has demonstrated circadian fluctuations in TJ proteins and highlighted the role of *Clock* and *Bmal1* in regulating TJ expression [34–37]. We identified several TJ genes and proteins, such as claudin-5, occludin, β-catenin, junctional adhesion molecules (JAM-2, JAM-3), and zonula occludens (ZO-1, ZO-2), as being influenced by circadian rhythms in endothelial cells [36,37], findings which were also confirmed in this study.

TJ proteins maintain the integrity of endothelial barrier function. Therefore, we also evaluated the impact of ZIKV and/or Bmal1 silencing on the functional integrity of endothelial cell monolayers. Even though ZIKV infected brain endothelial cells, this infection had no impact on transendothelial electrical resistance (TEER) (Figure 3B). While TEER has been frequently employed in barrier integrity studies, its values primarily reflect the resistance across the TJs of the cell monolayers. Therefore, it is not a fully reliable indicator for assessing barrier integrity [38]. In contrast, a significant increase in endothelial permeability in *Bmal1*-silenced and ZIKV-infected cultures was determined when barrier function was assessed by transendothelial passage of 20 KDa FITC-dextran (Figure 3C). Importantly, these changes were consistent with the downregulation of the expression of claudin-5, occludin, and ZO-1 proteins in endothelial cells subjected to ZIKV infections and *Bmal1* silencing. The *in vitro* data fully corroborated with animal experiments in which we observed that ZIKV infection downregulated the expression of claudin-5, occludin, JAM-3, and ZO-1 protein levels in Bmal1^ECKO^ mice (Figure 6).

The circadian system coordinates mitochondrial functions by providing critical cellular and tissue metabolic regulation [39,40]. Importantly, mitochondrial dysfunction is a powerful inducer of oxidative stress, which can compromise the integrity of TJs and the BBB [41–43]. Thus, we examined the contribution of circadian dysregulation and/or ZIKV infection on mitochondrial protein expression and bioenergetics. Silencing of *Bmal1* combined with ZIKV infection resulted in alternations of the expression of several mitochondrial proteins, including the pDRP1^s616^/DRP1 ratio, MFF, and OPA1 in brain endothelial cells (Figure 4). Moreover, we detected differential expression of the FIS1 protein level in brain microvessels of both WT and Bmal1^ECKO^ mice (Figure 7). These findings align with previous reports that mitochondrial fission-fusion dynamics and bioenergetics, particularly oxidative phosphorylation, are strongly influenced by circadian regulation [44]. The core circadian proteins were demonstrated to exhibit differential associations with mitochondrial dynamics and protein synthesis pathways in human muscle [45]. Studies have also documented the circadian control of mitochondria, including *in vitro* and *in vivo* models [46–48]. Importantly, the changes in the expression of the fission and fusion mitochondrial proteins were consistent with alterations of the expression of mitochondrial respiratory complex IV [49,50]. Our important findings indicated that deficiency of endothelial-specific Bmal1 markedly potentiated ZIKV-induced alterations of mitochondrial complexes II, III, and IV in brain microvessels.

ZIKV infection leads to mitochondrial failure, oxidative stress, and inflammatory overactivation [25,51,52]. In fact, ZIKV, similar to other flaviviruses, releases genetic material into the cytoplasm, where it associates with endoplasmic reticulum (ER) membranes to begin replication and synthesis of viral proteins within ER invaginations. ER alterations lead to a release of Ca2+, which can be taken up by mitochondria, leading to mitochondrial overload and failure [52]. ZIKV infection was also shown to induce mitochondrial fragmentation in human neural stem cells and the SNB-19 glioblastoma cell line. Furthermore, ZIKV infection-mediated changes in mitochondrial dynamics and mitophagy was described in trophoblast cells [24,25]. The findings of the present study are consistent with these observations, reinforcing the notion that disruption of mitochondrial function is a key aspect of ZIKV infection in vascular endothelium, which can be potentiated by circadian disruption.

## Conclusion

In conclusion, the present study demonstrates the significant impact of circadian dysregulation achieved by *Bmal1* silencing on the modulations of endothelial TJ proteins in ZIKV infection. Furthermore, the disruption of circadian rhythms potentiated alterations of mitochondrial dynamics in ZIKV-infected brain endothelium. Overall, the findings of this study may serve as a baseline for future efforts to better understand the implications of circadian rhythm disruption on the mechanisms of viral infections of an adult brain.

## Supplementary Material

1

## Data Availability

The datasets used and/or analysed during the current study are available from the corresponding author on reasonable request.

## List of abbreviations

ZIKV: Zika virus
CNS: central nervous system
BBB: blood-brain barrier
TJ: tight junction
PFU: plaque performing unit
FBS: fetal bovine serum
ATCC: American Type Culture Collection
EBM-2: Endothelial Cell Basal Medium-2
SDS: sodium dodecyl sulfate
BSA: bovine serum albumin
TEER: transendothelial electrical resistance
PFA: paraformaldehyde
Npas2: paralog neuronal PAS domain protein 2
JAMs: junctional adhesion molecule
ZOs: zona occludens
ER: endoplasmic reticulum

## References

[1] Posen HJ, Keystone JS, Gubbay JB, Morris SK. Epidemiology of Zika virus, 1947-2007. BMJ Glob Health [Internet]. 2016 [cited 2024 Mar 6];1:e000087. Available from: http://www.ncbi.nlm.nih.gov/pubmed/28588942 10.1136/bmjgh-2016-000087 PMC5321352 28588942

[2] Martins MM, Medronho RDA, Cunha AJLA Da. Zika virus in Brazil and worldwide: a narrative review. Paediatr Int Child Health [Internet]. 2021 [cited 2024 Mar 6];41:28–35. Available from: https://pubmed.ncbi.nlm.nih.gov/32576082/32576082 10.1080/20469047.2020.1776044

[3] Sharma V, Sharma M, Dhull D, Sharma Y, Kaushik S, Kaushik S. Zika virus: an emerging challenge to public health worldwide. Can J Microbiol [Internet]. 2020 [cited 2024 Mar 6];66:87–98. Available from: https://pubmed.ncbi.nlm.nih.gov/31682478/31682478 10.1139/cjm-2019-0331

[4] Singh RK, Dhama K, Khandia R, Munjal A, Karthik K, Tiwari R, Prevention and Control Strategies to Counter Zika Virus, a Special Focus on Intervention Approaches against Vector Mosquitoes—Current Updates. Front Microbiol [Internet]. 2018 [cited 2025 Jan 13];9:87. Available from: https://pmc.ncbi.nlm.nih.gov/articles/PMC5809424/29472902 10.3389/fmicb.2018.00087 PMC5809424

[5] Alimonti JB, Ribecco-Lutkiewicz M, Sodja C, Jezierski A, Stanimirovic DB, Liu Q, Zika virus crosses an in vitro human blood brain barrier model. Fluids Barriers CNS [Internet]. 2018 [cited 2024 Mar 6];15. Available from: https://pubmed.ncbi.nlm.nih.gov/29759080/10.1186/s12987-018-0100-y PMC5952854 29759080

[6] Chiu CF, Chu LW, Liao IC, Simanjuntak Y, Lin YL, Juan CC, The Mechanism of the Zika Virus Crossing the Placental Barrier and the Blood-Brain Barrier. Front Microbiol [Internet]. 2020 [cited 2020 Oct 13];11. Available from: https://pubmed.ncbi.nlm.nih.gov/32153526/10.3389/fmicb.2020.00214 PMC7044130 32153526

[7] Otani T, Furuse M. Tight Junction Structure and Function Revisited. Trends Cell Biol [Internet]. 2020 [cited 2024 Mar 6];30:805–17. Available from: https://pubmed.ncbi.nlm.nih.gov/32891490/32891490 10.1016/j.tcb.2020.08.004

[8] Egloff C, Fovet CM, Denis J, Pascal Q, Bossevot L, Luccantoni S, Fetal Zika virus inoculation in macaques revealed control of the fetal viral load during pregnancy. Virol J [Internet]. 2024 [cited 2024 Sep 13];21. Available from: https://pubmed.ncbi.nlm.nih.gov/39227837/10.1186/s12985-024-02468-x PMC11373269 39227837

[9] Auriti C, De Rose DU, Santisi A, Martini L, Piersigilli F, Bersani I, Pregnancy and viral infections: Mechanisms of fetal damage, diagnosis and prevention of neonatal adverse outcomes from cytomegalovirus to SARS-CoV-2 and Zika virus. Biochim Biophys Acta Mol Basis Dis [Internet]. 2021 [cited 2024 Sep 13];1867. Available from: https://pubmed.ncbi.nlm.nih.gov/34118406/10.1016/j.bbadis.2021.166198 PMC8883330 34118406

[10] Medina MT, Medina-Montoya M. New spectrum of the neurologic consequences of Zika. J Neurol Sci. 2017;383:214–5. 29108750 10.1016/j.jns.2017.10.046

[11] Muñoz LS, Parra B, Pardo CA. Neurological Implications of Zika Virus Infection in Adults. J Infect Dis [Internet]. 2017 [cited 2024 Mar 6];216:S897–905. Available from: https://pubmed.ncbi.nlm.nih.gov/29267923/29267923 10.1093/infdis/jix511 PMC5853915

[12] Zambrano LI, Fuentes-Barahona IC, Soto-Fernández RJ, Zuniga C, da Silva JC, Rodriguez-Morales AJ. Guillain-Barré syndrome associated with Zika virus infection in Honduras, 2016-2017. Int J Infect Dis [Internet]. 2019 [cited 2024 Mar 6];84:136–7. Available from: https://pubmed.ncbi.nlm.nih.gov/31096053/31096053 10.1016/j.ijid.2019.05.008

[13] Leda AR, Bertrand L, Andras IE, El-Hage N, Nair M, Toborek M. Selective Disruption of the Blood–Brain Barrier by Zika Virus. Front Microbiol [Internet]. 2019 [cited 2022 Aug 19];10. Available from: /pmc/articles/PMC6759472/10.3389/fmicb.2019.02158 PMC6759472 31620112

[14] Leiva S, Dizanzo MP, Fabbri C, Bugnon Valdano M, Luppo V, Levis S, Application of quantitative immunofluorescence assays to analyze the expression of cell contact proteins during Zika virus infections. Virus Res. 2021;304:198544. 34400226 10.1016/j.virusres.2021.198544

[15] Borrmann H, McKeating JA, Zhuang X. The Circadian Clock and Viral Infections. J Biol Rhythms [Internet]. 2021 [cited 2024 Mar 6];36:9–22. Available from: https://pubmed.ncbi.nlm.nih.gov/33161818/33161818 10.1177/0748730420967768 PMC7924106

[16] Blanco JR, Verdugo-Sivianes EM, Amiama A, Muñoz-Galván S. The circadian rhythm of viruses and its implications on susceptibility to infection. Expert Rev Anti Infect Ther [Internet]. 2022 [cited 2024 Mar 6];20:1109–17. Available from: https://pubmed.ncbi.nlm.nih.gov/35546444/35546444 10.1080/14787210.2022.2072296

[17] Schurhoff N, Toborek M. Circadian rhythms in the blood-brain barrier: impact on neurological disorders and stress responses. Mol Brain [Internet]. 2023 [cited 2024 Mar 6];16. Available from: https://pubmed.ncbi.nlm.nih.gov/36635730/10.1186/s13041-023-00997-0 PMC9835375 36635730

[18] Deaver JA, Eum SY, Toborek M. Circadian Disruption Changes Gut Microbiome Taxa and Functional Gene Composition. Front Microbiol [Internet]. 2018 [cited 2023 Apr 6];9:737. Available from: https://pubmed.ncbi.nlm.nih.gov/29706947/29706947 10.3389/fmicb.2018.00737 PMC5909328

[19] Zhuang X, Magri A, Hill M, Lai AG, Kumar A, Rambhatla SB, The circadian clock components BMAL1 and REV-ERBα regulate flavivirus replication. Nat Commun [Internet]. 2019 [cited 2024 Mar 6];10. Available from: https://pubmed.ncbi.nlm.nih.gov/30670689/10.1038/s41467-019-08299-7 PMC6343007 30670689

[20] de Lima Cavalcanti TYV, Lima MC, Bargi-Souza P, Franca RFO, Peliciari-Garcia RA. Zika Virus Infection Alters the Circadian Clock Expression in Human Neuronal Monolayer and Neurosphere Cultures. Cell Mol Neurobiol [Internet]. 2023 [cited 2024 Mar 6];44. Available from: https://pubmed.ncbi.nlm.nih.gov/38141078/10.1007/s10571-023-01445-2 PMC11407173 38141078

[21] Newman LE, Shadel GS. Mitochondrial DNA Release in Innate Immune Signaling. Annu Rev Biochem [Internet]. 2023 [cited 2024 Mar 6];92:299–332. Available from: https://pubmed.ncbi.nlm.nih.gov/37001140/37001140 10.1146/annurev-biochem-032620-104401 PMC11058562

[22] Banoth B, Cassel SL. Mitochondria in innate immune signaling. Transl Res [Internet]. 2018 [cited 2024 Mar 6];202:52–68. Available from: https://pubmed.ncbi.nlm.nih.gov/30165038/30165038 10.1016/j.trsl.2018.07.014 PMC6218307

[23] Freppel W, Roy M, Chatel-Chaix L. Flaviviridae and mitochondria: Everything you always wanted to know about their relationship but were afraid to ask. Virologie (Montrouge) [Internet]. 2022 [cited 2024 Mar 6]; Available from: https://pubmed.ncbi.nlm.nih.gov/35144917/10.1684/vir.2022.0926 35144917

[24] Lee JK, Shin OS. Zika virus modulates mitochondrial dynamics, mitophagy, and mitochondria-derived vesicles to facilitate viral replication in trophoblast cells. Front Immunol [Internet]. 2023 [cited 2024 Mar 6];14. Available from: https://pubmed.ncbi.nlm.nih.gov/37781396/10.3389/fimmu.2023.1203645 PMC10539660 37781396

[25] Yang S, Gorshkov K, Lee EM, Xu M, Cheng YS, Sun N, Zika Virus-Induced Neuronal Apoptosis via Increased Mitochondrial Fragmentation. Front Microbiol [Internet]. 2020 [cited 2024 Mar 6];11. Available from: https://pubmed.ncbi.nlm.nih.gov/33424801/10.3389/fmicb.2020.598203 PMC7785723 33424801

[26] Huang Y, Li Q, Kang L, Li B, Ye H, Duan X, Mitophagy Activation Targeting PINK1 Is an Effective Treatment to Inhibit Zika Virus Replication. ACS Infect Dis [Internet]. 2023 [cited 2024 Sep 13];9:1424–36. Available from: https://pubmed.ncbi.nlm.nih.gov/37300493/37300493 10.1021/acsinfecdis.3c00196

[27] Alger J, Buekens P, Cafferata ML, Alvarez Z, Berrueta M, Bock H, Microcephaly Outcomes among Zika Virus-Infected Pregnant Women in Honduras. Am J Trop Med Hyg [Internet]. 2021 [cited 2024 Sep 16];104:1737–40. Available from: https://pubmed.ncbi.nlm.nih.gov/33724927/33724927 10.4269/ajtmh.20-1483 PMC8103474

[28] DeBruyne JP, Weaver DR, Reppert SM. Peripheral circadian oscillators require CLOCK. Curr Biol. 2007;17. 10.1016/j.cub.2007.05.067 17637349

[29] Shi S, Hida A, McGuinness OP, Wasserman DH, Yamazaki S, Johnson CH. Circadian Clock Gene Bmal1 Is Not Essential After All; Functional Replacement with its Paralog, Bmal2. Curr Biol. 2010;20:316. 20153195 10.1016/j.cub.2009.12.034 PMC2907674

[30] Zoladek J, Legros V, Jeannin P, Chazal M, Pardigon N, Ceccaldi PE, Zika Virus Requires the Expression of Claudin-7 for Optimal Replication in Human Endothelial Cells. Front Microbiol [Internet]. 2021 [cited 2024 Sep 16];12:746589. Available from: /pmc/articles/PMC8488266/34616388 10.3389/fmicb.2021.746589 PMC8488266

[31] Miranda J, Martín-Tapia D, Valdespino-Vázquez Y, Alarcón L, Espejel-Nuñez A, Guzmán-Huerta M, Syncytiotrophoblast of Placentae from Women with Zika Virus Infection Has Altered Tight Junction Protein Expression and Increased Paracellular Permeability. Cells [Internet]. 2019 [cited 2024 Sep 16];8. Available from: https://pubmed.ncbi.nlm.nih.gov/31569528/10.3390/cells8101174 PMC6829373 31569528

[32] Kaur G, Pant P, Bhagat R, Seth P. Zika virus E protein modulates functions of human brain microvascular endothelial cells and astrocytes: implications on blood-brain barrier properties. Front Cell Neurosci [Internet]. 2023 [cited 2024 Sep 16];17:1173120. Available from: /pmc/articles/PMC10399241/37545876 10.3389/fncel.2023.1173120 PMC10399241

[33] de Almeida W, Deniz BF, Souza dos Santos A, Faustino AM, Ramires Junior OV, Schmitz F, Zika Virus affects neurobehavioral development, and causes oxidative stress associated to blood–brain barrier disruption in a rat model of congenital infection. Brain Behav Immun. 2023;112:29–41. 37146656 10.1016/j.bbi.2023.04.014

[34] Duraisamy SK, Srinivasan A, Sundar IK. House dust mite and Th2 cytokine-mediated epithelial barrier dysfunction attenuation by KL001 in 16-HBE cells. Tissue Barriers [Internet]. 2024 [cited 2024 Jul 8];12. Available from: https://pubmed.ncbi.nlm.nih.gov/37079442/10.1080/21688370.2023.2203841 PMC10832928 37079442

[35] Eum SY, Schurhoff N, Teglas T, Wolff G, Toborek M. Circadian disruption alters gut barrier integrity via a ß-catenin-MMP-related pathway. Mol Cell Biochem [Internet]. 2023 [cited 2024 May 22];478:581–95. Available from: https://pubmed.ncbi.nlm.nih.gov/35976519/35976519 10.1007/s11010-022-04536-8 PMC9938043

[36] Teglas T, Marcos AC, Torices S, Toborek M. Circadian control of polycyclic aromatic hydrocarbon-induced dysregulation of endothelial tight junctions and mitochondrial bioenergetics. Sci Total Environ [Internet]. 2024 [cited 2024 Sep 16];952. Available from: https://pubmed.ncbi.nlm.nih.gov/39218115/10.1016/j.scitotenv.2024.175886 PMC11444715 39218115

[37] Teglas T, Torices S, Taylor M, Coker D, Toborek M. Exposure to polychlorinated biphenyls selectively dysregulates endothelial circadian clock and endothelial toxicity. J Hazard Mater [Internet]. 2023 [cited 2024 May 22];454. Available from: https://pubmed.ncbi.nlm.nih.gov/37126901/10.1016/j.jhazmat.2023.131499 PMC10202419 37126901

[38] Mukherjee T, Squillantea E, Gillespieb M, Shao J. Transepithelial Electrical Resistance is Not a Reliable Measurement of the Caco-2 Monolayer Integrity in Transwell. Drug Deliv [Internet]. 2004 [cited 2024 Sep 16];11:11–8. Available from: https://www.tandfonline.com/doi/abs/10.1080/10717540490280345 15168786 10.1080/10717540490280345

[39] Casanova A, Wevers A, Navarro-Ledesma S, Pruimboom L. Mitochondria: It is all about energy. Front Physiol [Internet]. 2023 [cited 2024 Sep 16];14. Available from: https://pubmed.ncbi.nlm.nih.gov/37179826/10.3389/fphys.2023.1114231 PMC10167337 37179826

[40] Kim J, Sun W. Circadian coordination: understanding interplay between circadian clock and mitochondria. Anim Cells Syst (Seoul) [Internet]. 2024 [cited 2024 Sep 16];28:228–36. Available from: https://pubmed.ncbi.nlm.nih.gov/38721230/38721230 10.1080/19768354.2024.2347503 PMC11078072

[41] Yokoo K, Yamamoto Y, Suzuki T. Ammonia impairs tight junction barriers by inducing mitochondrial dysfunction in Caco-2 cells. FASEB J. 2021;35. 10.1096/fj.202100758R 34597422

[42] Srikantha P, Hasan Mohajeri M. The Possible Role of the Microbiota-Gut-Brain-Axis in Autism Spectrum Disorder. Int J Mol Sci. 2019;20. 10.3390/ijms20092115 PMC6539237 31035684

[43] Luciani A, Festa BP, Chen Z, Devuyst O. Defective autophagy degradation and abnormal tight junction-associated signaling drive epithelial dysfunction in cystinosis. Autophagy. 2018;14:1157–9. 29806776 10.1080/15548627.2018.1446625 PMC6103718

[44] Schmitt K, Grimm A, Dallmann R, Oettinghaus B, Restelli LM, Witzig M, Circadian Control of DRP1 Activity Regulates Mitochondrial Dynamics and Bioenergetics. Cell Metab [Internet]. 2018 [cited 2023 Dec 5];27:657–666.e5. Available from: https://pubmed.ncbi.nlm.nih.gov/29478834/29478834 10.1016/j.cmet.2018.01.011

[45] Figueroa-Toledo AM, Gutiérrez-Pino J, Carriel-Nesvara A, Marchese-Bittencourt M, Zbinden-Foncea H, Castro-Sepúlveda M. BMAL1 and CLOCK proteins exhibit differential association with mitochondrial dynamics, protein synthesis pathways and muscle strength in human muscle. J Physiol [Internet]. 2024 [cited 2024 Sep 16]; Available from: https://pubmed.ncbi.nlm.nih.gov/38922907/10.1113/JP285955 38922907

[46] Mezhnina V, Ebeigbe OP, Poe A, Kondratov R V. Circadian Control of Mitochondria in Reactive Oxygen Species Homeostasis. Antioxid Redox Signal [Internet]. 2022 [cited 2024 Sep 16];37:647–63. Available from: https://pubmed.ncbi.nlm.nih.gov/35072523/35072523 10.1089/ars.2021.0274 PMC9587791

[47] Jin Z, Ji Y, Su W, Zhou L, Wu X, Gao L, The role of circadian clock-controlled mitochondrial dynamics in diabetic cardiomyopathy. Front Immunol [Internet]. 2023 [cited 2024 Sep 16];14. Available from: https://pubmed.ncbi.nlm.nih.gov/37215098/10.3389/fimmu.2023.1142512 PMC10196400 37215098

[48] De Goede P, Wefers J, Brombacher EC, Schrauwen P, Kalsbeek A. Circadian rhythms in mitochondrial respiration. J Mol Endocrinol [Internet]. 2018 [cited 2023 Dec 7];60:R115–30. Available from: https://pubmed.ncbi.nlm.nih.gov/29378772/29378772 10.1530/JME-17-0196 PMC5854864

[49] Hsieh YC, Yu HP, Suzuki T, Choudhry MA, Schwacha MG, Bland KI, Upregulation of mitochondrial respiratory complex IV by estrogen receptor-β is critical for inhibiting mitochondrial apoptotic signaling and restoring cardiac functions following trauma-hemorrhage. J Mol Cell Cardiol [Internet]. 2006 [cited 2024 Sep 16];41:511–21. Available from: http://www.jmcc-online.com/article/S002228280600589X/fulltext 16859701 10.1016/j.yjmcc.2006.06.001

[50] Havlíčková Karbanová V, Čížková Vrbacká A, Hejzlarová K, Nůsková H, Stránecký V, Potocká A, Compensatory upregulation of respiratory chain complexes III and IV in isolated deficiency of ATP synthase due to TMEM70 mutation. Biochim Biophys Acta [Internet]. 2012 [cited 2024 Sep 16];1817:1037–43. Available from: https://pubmed.ncbi.nlm.nih.gov/22433607/22433607 10.1016/j.bbabio.2012.03.004

[51] García CC, Vázquez CA, Giovannoni F, Russo CA, Cordo SM, Alaimo A, Cellular Organelles Reorganization During Zika Virus Infection of Human Cells. Front Microbiol [Internet]. 2020 [cited 2024 Nov 26];11. Available from: https://pubmed.ncbi.nlm.nih.gov/32774331/10.3389/fmicb.2020.01558 PMC7381349 32774331

[52] Ledur PF, Karmirian K, Pedrosa C da SG, Souza LRQ, Assis-de-Lemos G, Martins TM, Zika virus infection leads to mitochondrial failure, oxidative stress and DNA damage in human iPSC-derived astrocytes. Scientific Reports 2020 10:1 [Internet]. 2020 [cited 2024 Nov 26];10:1–14. Available from: https://www.nature.com/articles/s41598-020- 57914-x 10.1038/s41598-020-57914-x PMC6985105 31988337

