# Supplementary material for: Circadian misalignment potentiates blood-brain barrier disruption and mitochondria dysregulation in Zika virus infection
Source: Res Sq. 2025 Apr 22:rs.3.rs-6298126. Preprint. [Version 1] doi: 10.21203/rs.3.rs-6298126/v1 (PMC12045451; doi:10.21203/rs.3.rs-6298126/v1)
Supplement: 1 [file NIHPPrs6298126v1-supplement-1.pdf]

Supplementary Data

Supplementary Table 2. List of antibodies used in immunoblotting.

Target protein	Company	Clone	Catalog#
Bmal1	Cell Signaling	monoclonal	#14020S
Claudin-5	Abcam, ThermoFisher	monoclonal, polyclonal	#ab131259, #34-1600
β-catenin	Abcam	monoclonal	#ab16051
Drp1	Cell Signaling	monoclonal	#14647
GAPDH	ThermoFisher	monoclonal	#MA5-15738-D680
Jam-2	Abcam	monoclonal	#ab156586
Jam-3	ThermoFisher	polyclonal	#40-9000
Mff	Cell Signaling	Monoclonal	#84580
Fis1	ThermoFisher	monoclonal	#MA5-27836
Occludin	Invitrogen, Cell Signaling	monoclonal	#33-1500, #91131
Opa1	Cell Signaling	monoclonal	#80471
Total Oxphos cocktail (rodent)	Abcam	monoclonal	#ab110413
Total Oxphos cocktail (human)	Abcam	monoclonal	#ab110411
DRP1	Cell Signaling	monoclonal	#4494
pDRP1(ser616)	Cell Signaling	monoclonal	#14647
β-tubulin	Thermo Fisher	monoclonal	#MA5-16308-D680
Tom20	Cell Signaling	Monoclonal	#42406
ZO-1	Invitrogen	monoclonal	#33-9100
ZO-2	Invitrogen	polyclonal	#711400
IRDye® 800CW Goat Anti-Mouse IgG	LICOR	monoclonal	#926-32210
IRDye® 680RD Goat anti-Mouse IgG Secondary Antibody	LICOR	monoclonal	#926-68070
IRDye® 800CW Goat anti-Rabbit IgG Secondary Antibody	LICOR	monoclonal	#926-32211
IRDye® 680RD Goat anti-Rabbit IgG Secondary Antibody	LICOR	monoclonal	#926-68071

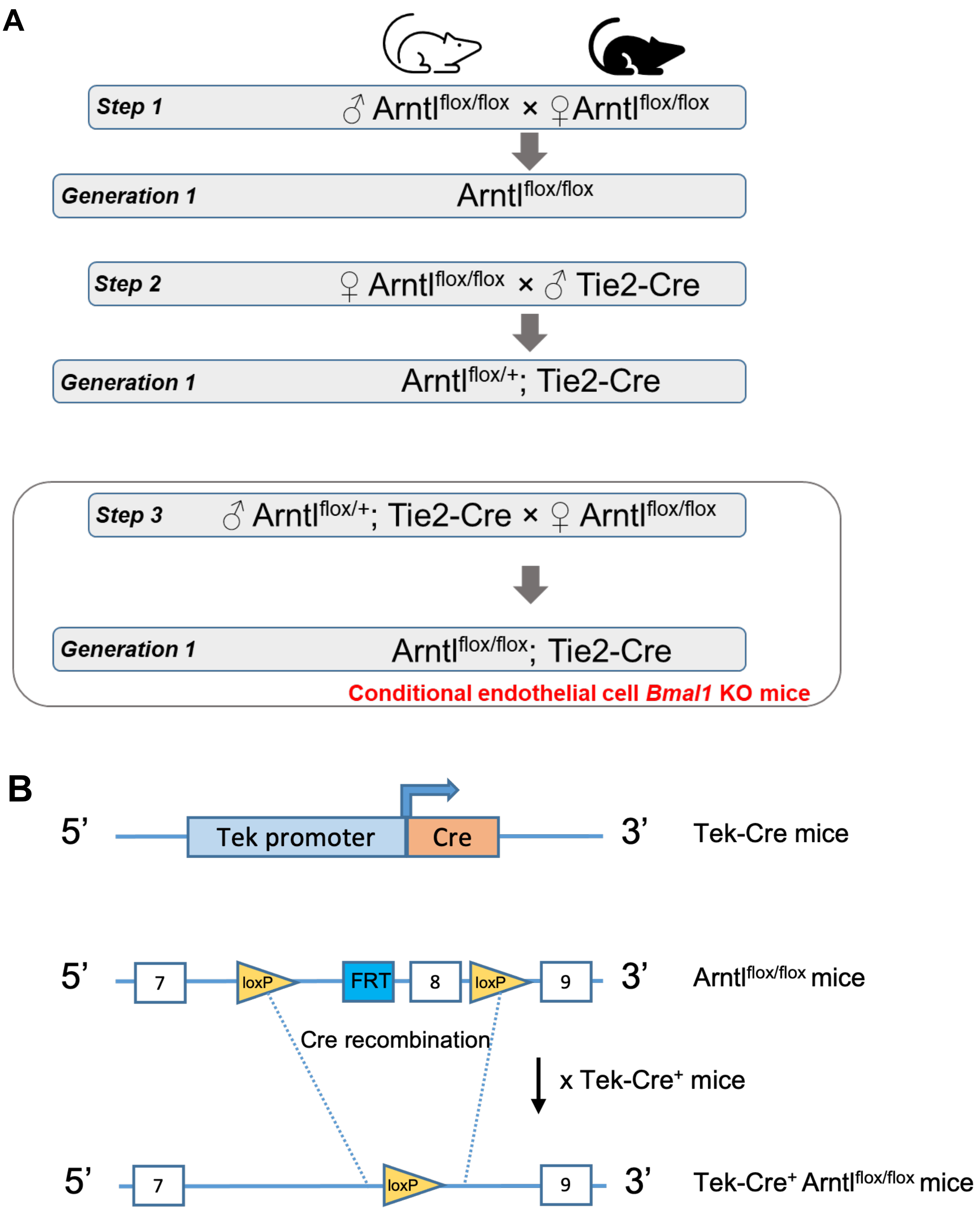

Supplementary Figure 1. Breeding of Bmal1^{ECKO} mice. (**A**) Breeding for Bmal1$^{flox/flox}$; Tek-Cre mice. (**B**) The Bmal1$^{flox/flox}$ and Tek-Cre mice were bred via a Cre recombination-based system.
